# Live Bird Exposure among the General Public, Guangzhou, China, May 2013

**DOI:** 10.1371/journal.pone.0143582

**Published:** 2015-12-01

**Authors:** Qiuyan Liao, Jun Yuan, Eric H. Y. Lau, Guang Yan Chen, Zhi Cong Yang, Xiao Wei Ma, Jian Dong Chen, Yan Hui Liu, Chang Wang, Xiao Ping Tang, Yu Fei Liu, Li Zhuo, Gabriel M. Leung, Wei Zhang, Benjamin J. Cowling, Ming Wang, Richard Fielding

**Affiliations:** 1 School of Public Health, the University of Hong Kong, Hong Kong, People’s Republic of China; 2 Guangzhou Center for Disease Control and Prevention, Guangzhou, People’s Republic of China; 3 Panyu Center for Disease Control and Prevention, Guangzhou, People’s Republic of China; 4 The Eighth People’s Hospital of Guangzhou, Guangzhou, People’s Republic of China; 5 Guangdong Center for Disease Control and Prevention, Guangzhou, People’s Republic of China; Shanxi University, CHINA

## Abstract

**Background:**

A novel avian-origin influenza A(H7N9) caused a major outbreak in Mainland China in early 2013. Exposure to live poultry was believed to be the major route of infection. There are limited data on how the general public changes their practices regarding live poultry exposure in response to the early outbreak of this novel influenza and the frequency of population exposure to live poultry in different areas of China.

**Methodology:**

This study investigated population exposures to live birds from various sources during the outbreak of H7N9 in Guangzhou city, China in 2013 and compared them with those observed during the 2006 influenza A(H5N1) outbreak. Adults were telephone-interviewed using two-stage sampling, stratified by three residential areas of Guangzhou: urban areas and two semi-rural areas in one of which (Zengcheng) A(H7N9) virus was detected in a chicken from wet markets. Logistic regression models were built to describe practices protecting against avian influenza, weighted by age and gender, and then compare these practices across residential areas in 2013 with those from a comparable 2006 survey.

**Principal Findings:**

Of 1196 respondents, 45% visited wet markets at least daily and 22.0% reported buying live birds from wet markets at least weekly in April-May, 2013, after the H7N9 epidemic was officially declared in late March 2013. Of those buying live birds, 32.3% reported touching birds when buying and 13.7% would slaughter the poultry at home. Although only 10.1% of the respondents reported raising backyard birds, 92.1% of those who did so had physical contact with the birds they raised. Zengcheng respondents were less likely to report buying live birds from wet markets, but more likely to buy from other sources when compared to urban respondents. Compared with the 2006 survey, the prevalence of buying live birds from wet markets, touching when buying and slaughtering birds at home had substantially declined in the 2013 survey.

**Conclusion/Significance:**

Although population exposures to live poultry were substantially fewer in 2013 compared to 2006, wet markets and backyard poultry remained the two major sources of live bird exposures for the public in Guangzhou in 2013. Zengcheng residents seemed to have reduced buying live birds from wet markets but not from other sources in response to the detection of H7N9 virus in wet markets.

## Introduction

A novel avian-origin influenza A(H7N9) virus emerged in early 2013 causing more than 100 human infections in eastern China between February and May 2013 [1–3]. Estimates of fatality risk for patients admitted to hospital indicated that H7N9 virus infection was more severe than seasonal influenza but less so than A(H5N1) infection [2]. Recent exposure to live poultry was reported by the majority of laboratory-confirmed H7N9 cases and is thought to constitute the major route of infection [2,3], but there is good evidence that human-to-human transmissibility remains very low [4]. Temporary closure of live poultry markets (LPMs) in major cities in eastern China was associated with rapid reduction in incidence of confirmed human H7N9 cases [5,6]. However, while temporary closure of LPMs for market rest days received strong support from the public, public support for permanent closure of LPMs remained low in China [7,8]. Currently, there are limited data on the frequency of population exposures to live poultry in different areas of China [7].

Guangzhou, the provincial capital of Guangdong province in Southern China, first identified H7N9 virus in chicken from LPM surveillance in May 2013, but the first human case of H7N9 infection did not emerge until January 2014 [9]. A previous survey conducted in April 2013 indicated that people in Guangzhou reported the highest rate of exposure to live poultry among the five selected cities in Mainland China [7]. However, it is unknown whether the first notification of H7N9 virus in LPMs would prompt behavioural change that reduces exposure to live poultry and whether such changes are specific to LPMs but not other sources. Therefore, we conducted this population-based survey in early June 2013 to investigate public risk perception in Guangzhou relating to H7N9 and exposure to live birds from different sources. A brief report on public attitudes, perceptions and changes in household buying of live poultry from wet markets has been published [10]. In this paper, we reported detailed information on personal exposure to live birds from wet markets, backyards and other sources, and adoption of protective measures during contact with live birds in the first wave of the H7N9 epidemic (between April and May 2013). Although there was no human infection of H7N9 in Guangzhou during this period, the detailed assessment of population behaviours regarding live poultry exposure from difference sources and associated protective behaviours during this early epidemic stage should be able to assess the effectiveness of risk communication of the Chinese Government since the outbreak of H7N9 in March 2013. Furthermore, this early assessment should also provide important baseline information for future assessment of population risk of H7N9 infection.

It has been previously reported that exposure to live birds differs by residential, specifically urban or rural, setting [7,11], with urban residents’ main route of exposure to live poultry being from LPMs while for rural residents’ exposure being mainly from backyard poultry husbandry [7,11]. Urban and rural residents also reportedly differ in adherence to protective behaviours during buying and handling of poultry, with rural residents being less likely to adopt protective behaviours [11]. Moreover, we have previously reported that change in live poultry exposure apparently differs by geographical proximity to the epidemic, with those residents living closer to an epidemic area being more likely to change their risky behaviours [12]. This fluidity in behavioural change may also reshape the poultry trading network or flow and subsequently the avian and human infection risk in proximal regions. Therefore, the second objective of this study was to compare personal exposure to live birds from various sources and their associated protective behaviours between urban, non-epidemic rural areas and epidemic rural area (Zengcheng where H7N9 infection was detected in a live chicken from wet markets). If confirmed, this could inform more targeted public health interventions for reducing population risk of avian influenza infection.

Between March and June 2006 a population-based survey was conducted in Guangzhou city to explore public habits regarding buying live birds from wet markets, bird handling at home and backyard bird raising prior to the H5N1 outbreak, related H5N1 influenza risk perceptions [11,13] and changes in bird-buying habits following the H5N1 epidemic (S1 Fig). This survey involved 1,550 adults (including 1363, 98 and 89 residents from the urban areas, Conghua and Zengcheng, respectively) aged 18 or above completing face-to-face interviews and is described elsewhere [11,13]. The 2006 survey was conducted during an H5N1 outbreak in China and immediately following the report of the first laboratory-confirmed H5N1 human case in Guangzhou in February 2006. At that time, H5N1 was the first widely recognized emerging highly pathogenic strain of avian influenza identified in China that could transmit from birds to human and thereby was perceived to pose a new threat to human health. Subsequent to H5N1, other avian influenza viruses have been identified from LPMs but H7N9 is the only other one found to date that is known to be transmissible to humans and have severe clinical consequences. The repeated identification of avian influenza viruses from LPMs is likely to prompt reduction in risky practices of buying and handling of live poultry among the public as a consequence of enhanced population risk perception or policy changes that reduce live poultry availability [14]. However, it may also lead to warning or risk fatigue, which occurs following repeated threat warnings that are widely perceived to have failed to materialize, and which results in the resumption of normal habits of buying and handling of poultry. Therefore, this study also compared public exposures to live poultry and risk perceptions of avian influenza with those in the 2006 survey [11,13].

## Methods

The administrative divisions of Guangzhou consists of ten urban districts and two adjacent satellite county-level cities (Conghua and Zengcheng) with telephone land-line penetration exceeding 90% in all of these areas [15]. To recruit subjects, sampling was first stratified by three residential areas: urban districts, Conghua and Zengcheng, to represent areas with different level of geographical closeness to the confirmed H7N9 infection in a chicken. Prevalence of buying live poultry among the general public in the urban and semi-rural areas was estimated to be 70–80% [11]. We estimated that a sample size of around 300 for each area would be sufficient to examine such population prevalence in each area with a 95% confidence interval and a marginal error of 0.05 [16]. A sample size of 300 in each area was also sufficient to detect a 10% prevalence difference between areas with a 95% confidence interval and power of 80% [16]. Since over 80% of the Guangzhou population resided in the ten urban districts of Guangzhou [15], this study oversampled respondents in the urban districts. Mitofsky–Waksberg two-stage sampling [17] was performed to select households. This is a clustered sampling approach refined by Warren Mitofsky and Joseph Waksberg based on random digit dialing sampling to improve the efficiency of selecting a random sample of eligible household telephone numbers. In the first stage, a total of 120, 60 and 60 telephone prefixes in urban districts, Conghua and Zengcheng, respectively, were randomly selected. In the second stage, telephone numbers were randomly generated and called to recruit five households for each prefix. Within each household, one subject aged above 18 years and whose birthday was closest to the interview date was invited for the telephone interview based on a standardized questionnaire. To improve response rate, a prepaid card for phone recharge valued at US$1.63 was given to each subject who successfully completed the interview. All interviews were conducted from 18:30 to 21:30 between May 30, 2013 and June 7, 2013. The study obtained ethical approval from the Human Research Ethics committees of Guangzhou Center for Disease Control and Prevention. Since telephone interview was used to recruit subjects and to complete the questionnaire, written informed consent could not be obtained and thereby was waived by the ethics committees but verbal consent from each participant was required. All participants gave verbal consent after a brief introduction about the study purposes which was recorded by the interviewers before the formal telephone interview started.

We used a questionnaire similar to that used in the previous study in 2006 [11,13] which was pretested for length, content validity and comprehensibility. The final questionnaire took around 5–10 minutes for each interview. Most questions collected information on behaviors related to live bird exposures in April and May (after government announced the H7N9 outbreak in late March) [18]. Here “live bird exposure” was used to refer to both exposure to live poultry such as chicken, ducks and geese, and other birds such as quails, pet birds and wide birds. Information gathered included visits to wet markets with live birds (live poultry trading markets including whole sale, retail and mixed poultry markets), buying live birds from wet markets and from other places, raising backyard birds, touching and killing birds, contact with sick birds, wild birds or bird droppings, and associated protective measures. Respondents were specifically asked whether they had bought less or even stopped buying live poultry over the past two months, and reasons for such changes. Demographics of the respondents were also recorded. Details about the relevant study measures of this paper are presented in S1 Table, S1 and S2 datasets.

Proportions standardized by age, gender and education attainment, and the corresponding 95% confidence intervals (95% CI) were calculated using logistic regression models for each type of behavior in the three residential areas and the total sample.The logistic regression model used for calculating the risk ratios of each behavioural outcome is showed below:

$$logit\;(\text{Pr}(Y=1))=b_{0}+b_{1}age_{1}+b_{2}age_{2}+b_{3}e{du}_{1}+b_{4}{edu}_{2}+b_{5}gender$$ (1)

In Eq (1), Y is the behavioral outcome of interest while b_0_ is the intercept and b_1_-b_5_ are the coefficients for the corresponding confounders. Based on Eq (1), the proportion of each behavioural outcome was predicted using marginal standardization [19] and weighted by population age and gender.When the behavioral outcomes were calculated for each area, a random effect of the residential areas was included in the logistic regression model to account for the variability of the outcome variables between the semi-rural and the urban areas. Logistic regression models were also conducted to calculate and compare proportions of common behaviours and perceptions relating to live bird exposure and avian influenza in the 2006 survey and the current survey. The proportions calculated were weighted by population age and gender in Guangzhou [20] for comparison across areas and across the two surveys. The same conservative methods used in the previous survey [11] were again applied to calculate the rate of touch when buying on the basis of touching frequency (e.g., "always", "usually", "sometimes" and "never" were quantified at an approximate rate of "1", "0.67", "0.25" and "0", respectively) in the current survey and compared with that of 2006. P-values of less than 0.05 were considered to be statistically significant. All statistical analyses were conducted using STATA software (version 10.0; STATA Corp., College Station, TX).

## Results

### The participants

A total of 1196 subjects completed the interview from 1930 eligible calls, a response rate of 62.0%. Of the 1196 subjects, 19 (1.6%) were excluded due to missing information on age, leaving 1177 subjects for subsequent data analysis. Comparison of the characteristics of respondents from each residential area and the total sample was previously described [10]. Based on Pearson chi-square test, the samples from urban districts, Conghua and Zengcheng did not differ by gender, age or marital status but significantly differed by education attainment and personal income, with respondents from urban districts reporting higher education attainment and personal income [10]. The total sample were generally representative of the Guangzhou general population except that the sample were slightly better educated and more likely to be married or formerly married [10].

### Live bird exposure

Of the respondents, 45.0% (95% CI: 41.0%-49.1%) visited wet markets where live birds were sold "daily or more" after the government announced the H7N9 outbreak in China (Table 1). Visiting wet markets did not differ by residential areas. Around 22.0% (95% CI: 18.8%-25.5%) of the respondents reported buying live birds from wet markets "at least weekly" (Table 1). The proportion of personally buying live birds from wet markets was significantly lower in Zengcheng (relative to urban) but was comparable between the urban area and Conghua. A small proportion (2.3%, 95% CI: 1.5–3.7%) of the respondents bought live birds from poultry farms or small backyard holders (Table 1). Buying live birds from these sources was significantly more prevalent in Zengcheng and Conghua than in urban districts.

**Table 1:** Behaviours related to live bird^a^ exposure and associated protective measures, Guangzhou, 2013^b^.

Behaviours	Urban (%, 95% CI) N = 594	Conghua (%, 95% CI) N = 283	Zengcheng (%, 95% CI) N = 300	Overall (%, 95% CI) N = 1177
**Exposure**				
Visiting wet markets over the past two months				
At least monthly but less than weekly	6.9 (4.8–9.8)	10.9 (7.2–16.3)	4.4 (2.6–7.5)	6.2 (4.5–8.5)
At least weekly but less than daily	24.7 (20.8–29.1)	21.1 (16.0–27.4)	24.1 (19.0–30.0)	24.2 (20.9–27.9)
At least daily	44.7 (40.1–49.4)	42.3 (35.4–49.5)	40.4 (34.3–46.8)	45.0 (41.0–49.1)
Respondents buying live birds from wet markets				
At least monthly but less than weekly	16.8 (13.7–20.5)	22.6 (17.6–28.6)	17.0 (12.5–22.7)	17.3 (14.5–20.5)
At least weekly	22.8 (19.1–27.1)	18.0 (13.5–23.7)	10.7 (7.5–14.9)^c^	22.0 (18.8–25.5)
Buying live birds from poultry farms or backyard poultry holders	1.8 (0.9–3.8)	6.4 (4.1–9.8)^c^	5.8 (3.4–9.6)^c^	2.3 (1.5–3.7)
Of those buying, touch when buying	31.5 (25.2–38.6)	37.4 (28.4–47.3)	49.1 (36.8–61.5)^c^	32.2 (26.5–38.4)
Of those buying, slaughter birds at home	12.4 (8.1–18.4)	25.7 (18.2–34.9)^c^	10.3 (5.4–18.8)	13.7 (9.9–18.8)
Raising backyard birds	8.8 (6.3–12.1)	30.6 (24.0–38.1)^c^	20.3 (15.2–26.6)^c^	10.1 (7.9–12.1)
Of those raising, touching^d^	89.5 (75.1–96.0)	87.8 (76.1–94.2)	91.8 (78.4–97.2)	92.1 (86.1–95.6)
Contact with live birds or their droppings outdoors	7.8 (5.7–10.7)	14.2 (10.3–19.2)^c^	10.0 (6.8–14.5)	7.9 (6.1–10.1)
Contact with sick/dead birds^e^	-	-	-	0.1 (0.0–0.4)
**Associated protective measures**				
Frequent washing or disinfecting hands				
Of those touching birds when buying	52.5 (38.5–66.1)	60.2 (42.8–75.4)	41.0 (23.5–61.2)	50.1 (37.2–62.9)
Of those touching backyard birds, bird cages or droppings	81.5 (57.0–93.6)	88.9 (76.9–95.0)	85.2 (67.7–94.0)	84.6 (70.6–92.6)
Of those killing poultry at home^e^				
Wear gloves	-	-	-	0.9 (0.4–2.3)
Wear a face mask	-	-	-	0.0 (0.0–0.2)
Wear aprons/outer garments/coveralls	-	-	-	13.7 (6.2–27.3)
Wear boots/boot covers	-	-	-	0.8 (0.2–3.4)
**Change of live bird buying behaviour due to H7N9 epidemic**				
Bought less	39.3 (34.8–43.9)	45.8 (38.9–52.9)	39.7 (33.4–36.4)	39.6 (35.9–43.6)
Stopped buying	19.0 (15.6–22.9)	9.8 (6.4–14.6)^c^	30.8 (25.3–37.0)^c^	19.0 (16.1–22.2)

^a^Including chickens, ducks, geese and other birds.

^b^All values in the table are percentage weighted by population age and gender and adjusted by sample age, gender and education obtainment.

^c^Behaviours were significantly different from those in the urban districts (p<0.05).

^d^Including touching the birds, bird cages or droppings.

^e^Numbers of respondents who gave affirmative responses in the variables were too small to enable reliable estimates by residential areas.

Of those buying live birds, 32.3% (95% CI: 26.5–38.4%) reported touching birds when buying and 13.7% (95% CI: 9.9–18.8%) reportedly slaughtered the poultry at home (Table 1). A significantly higher proportion of Zengcheng respondents touched the birds when buying while significantly higher proportion of Conghua respondents slaughtered birds at home compared to urban respondents (Table 1). Around 10.1% (95% CI: 7.9%-12.1%) of the sampled families raised backyard birds and most of these respondents reported touching backyard birds, bird cages or droppings at home; the prevalence of raising backyard birds was higher in Zengcheng and Conghua than in urban districts. Conghua respondents were more likely to report having had contact with birds or bird droppings outdoors relative to urban districts respondents (Table 1).

50.1% of the respondents reported cleaning hands immediately after touching birds when buying and most say that they do this after touching backyard birds, bird cages or bird droppings. When slaughtering birds at home, the most frequently adopted protective measure was wearing aprons, outer garments or coveralls (13.7%, 95% CI: 6.2–27.3%), though a few reportedly wore gloves, boots or face masks (Table 1).

Of the 1177 respondents, 39.6% (95% CI: 35.9–43.6%) reported reducing buying and 19.0% (95% CI: 16.1–22.2%) stopping buying live poultry due to the H7N9 epidemic (Table 1). Compared with urban residents, Zengcheng residents were more likely to report stopping buying while Conghua residents were less likely to report stopping buying live birds. Among the 720 respondents who reported reducing or stopping buying live birds from wet markets, the predominant reasons were to avoid "avian flu" (either H7N9 specific or not) (85.7%, 95% CI: 82.9–88.2%) and "health concerns" (33.2%, 95% CI: 29.7–36.8%)

### Comparison of behaviours and perceptions relating to live bird exposure and avian influenza between 2006 and 2013

**Fig 1:**
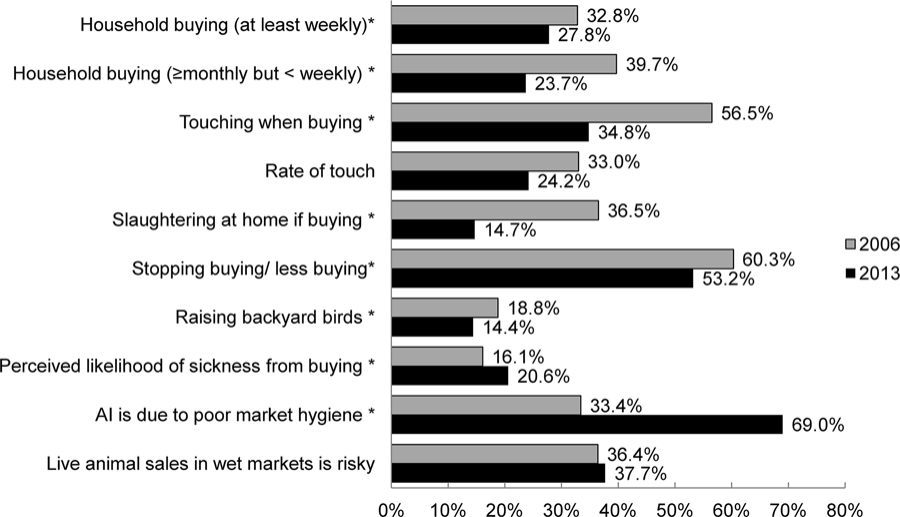
Comparison of perceptions and practices relating to live bird exposure and wet markets between 2006 and 2013. ^*^Behaviours or perceptions were significantly different (p<0.05) between year 2006 and 2013. AI: Avian influenza. All percentages were adjusted by age, gender and education attainment and weighted by population age and gender.

After adjusting for age, gender, education attainment and weighted by population age and gender [20], compared to the usual habits measured in 2006, there were significantly smaller proportions of households raised backyard birds (18.8% in 2006 vs.14.4% in 2013), bought live birds from wet markets (32.8% in 2006 vs. 27.8% in 2013 for buying "at least weekly"), touched when buying (56.6% in 2006 vs. 34.8% in 2013), or slaughtered birds at home if buying (36.5% in 2006 vs. 14.7% in 2013) in 2013; however, significantly fewer respondents in 2013 reportedly reduced or stopped buying live birds in response to the H7N9 epidemic than those in response to the H5N1 epidemic in 2006 (Fig 1). Similar proportions of respondents perceived that "live animal sales in wet markets are risky" in the two surveys but significantly more respondents perceived that they or their family were "likely/very likely" to get sickness due to buying live birds from wet markets and that "avian influenza is due to poor market hygiene" in the 2013 survey (Fig 1). In the current survey, the estimated rate of touching when buying [11] was 24.2%, around a 27% abosulute reduction compared to the touch rate of 33% in the 2006 survey.

## Discussion

During the H7N9 epidemic in mid 2013 after H7N9 outbreak was announced by the Chinese government, we observed a high prevalence of frequent wet markets visits in the population, suggesting that these remain an important resource for buying food among both urban and rural Guangzhou residents. Compared with the usual habits of live poultry-related practices recorded in the 2006 survey [11], the Guangzhou public has substantially reduced their rates of buying live birds from wet markets, touching when buying, slaughtering birds at home if buying and raising backyard birds. The reduction of these risky practices was accompanied by enhanced perceived risk of avian influenza infection linking to live birds in wet markets. These changes may reflect the impact of the H7N9 epidemic in China, or may reflect a long-term cultural trend influenced by repeated poultry-related epidemics, as well as changes in consumer preferences. The reduction in buying behavior may also be a temporary change [21] in reaction to the present epidemic, but if extensive, this may have helped to mute future H7N9 epidemic waves through reducing poultry movements in south and south-east China [22].

Reported declines in buying frequency (including stopping) were found to be greater in 2006 in response to the H5N1 epidemic. The 2006 survey was conducted after a total of 16 human H5N1 cases were successively reported from 10 provinces in Mainland China between November 2005 and March 2006 [23] including one case in Guangzhou. The greater geographic proximity, and the relative novelty of the H5N1 epidemic in Guangzhou were likely to prompt the greater behavioural change in 2006. However, perceived likelihood of contracting H5N1 virus infection was lower than that of contracting H7N9 virus infection from buying live birds. Although only poultry sources of avian H7N9 infection were identified in Guangzhou, a total of 131 laboratory-confirmed H7N9 cases have been reported in other provinces in Mainland China between March and May 2013 [2]. The greater number of H7N9 human cases reported in a relatively short period may have led to the greater perceived likelihood of contacting H7N9 infection from buying live birds.

Despite substantial reductions in buying frequency, 22% of respondents continued buying live birds from wet market at least weekly. This prevalence if extrapolated to the whole population of Guangzhou (12.7 million people) [20], would result in at least 2,794,000 persons buying live birds from wet markets per week in Guangzhou. Taking into account a touch rate of 24%, this would generate approximately 670,000 human-bird contacts per week in the Guangzhou population. Besides retailed birds, backyard birds are also another important source of live poultry exposure for the public [12]. Although the prevalence of raising backyard birds was relative low (10%) in our sample, the prevalence of touching backyard birds was much higher (92%) than touching when buying. Touching backyard birds is most likely to occur daily. If this extrapolates to the overall 3,684,370 households (each consists of 2.73 persons on average) of Guangzhou, it would result into 925,000 person-exposures to backyard birds per day in Guangzhou, probably concentrated mainly in the rural than that in the urban districts. This indicates that exposure to domestic birds remains high and greatly exceeded live bird exposures from wet markets. However, H7N9 infection rates among retail and backyard birds remain unknown in Guangzhou limiting further quantification of the population risk from these sources.

Only half of those who bought live poultry touched the birds directly when buying, but more than 80% of these respondents reported cleaning their hands immediately after touching birds in the backyard, probably because hand-washing facilities are more likely to be available at home than in the LPMs. Easy access to hand-washing facilities in wet markets may promote handwashing practices for those who touch birds. However, persons who reported slaughtering birds at home generally adopted no protective measures, an practice similar to that observed among live poultry traders[24]. This risks exposing them and their families to avian-sourced infections like H7N9 and gives a direction for educational interventions.

Compared to other residential areas more Zengcheng respondents stopped buying live birds from wet markets in response to the identification of H7N9 virus in a chicken in Zengcheng, suggesting that geographic proximity to outbreak prompts prominent behavioral change [12,25]. This may reshape the trading pattern in the cities around Zengcheng. For example, traders in Zengcheng may seek nearby markets, such as Conghua and other cities around, for selling their poultry, or reduce the price of the unsold poultry to minimize their economic loss. These changes in trading pattern are likely to spread risk of H7N9 infection to other areas. Furthermore, the reduction in buying poultry from LPMs among people in the epidemic area could increase viral amplification through longer transportation or stay in wholesale or retail markets. Despite reduction in buying rate, among those buying live poultry, the proportion of respondents having at least some direct contact with the birds they bought was significantly greater in Zengcheng than that in the urban districts, suggesting that people in Zengcheng who resist changing their habit of buying live birds from wet markets may also persist in touching birds when buying. Except for reduced frequency of buying live birds from wet markets, Zengcheng residents did not otherwise reduce their exposure to live poultry from other sources, suggesting that they did not extrapolate the risk from wet markets to retail sales of birds generally. Compared with urban residents, rural residents likely have higher live bird exposures due to more prevalent practices of buying live birds from poultry farms or backyard poultry holders directly, touching birds when buying, slaughtering birds at home, raising backyard birds and having contact with wild birds or bird droppings outdoors. Although LPMs were the direct sources of H7N9 infection for human, previous studies suggested that wild birds were the important original source of H7N9 infection [26, 27]. This suggests that risk of H7N9 infection in rural residents may not reduce as much as their urban counterparts when LPMs are temporarily closed due to H7N9 outbreaks, especially most domestic birds in rural China are raised with limited bio-security (have close contact with wild birds) [28]. Conghua residents were least likely to stop buying live birds from wet markets, suggesting that rural residents are more likely to resist changes in their existing behaviours especially if no local epidemic occurs possibly because of risk fatigue or greater familiarity with poultry breeding creates indifference to their being a potential source of infection [29].

Live animal markets were found to be responsible for the spread of Severe Acute Respiratory Syndrome Coronavirus in 2003 [30], and many H5N1 avian influenza virus infections [31]. However, perceived health risk from live animal sales did not increase from 2006 through 2013 even though human H7N9 cases were again closely linked with live bird exposures in wet markets [2,3]. This suggests that knowledge deficiency or risk fatigue and adaptation may both be targets for public health education. However, compared with the 2006 survey, the proportion of respondents citing poor market hygiene as a cause of avian influenza infection had doubled in 2013, suggesting increased awareness of and attribution to poor hygiene rather than retail practice s that artificially concentrate and mix live animals as the main cause.

There are some limitations in our study. First, all data were self-reported making results potentially subject to social desirability bias. Second, volunteer subjects were recruited and potential selection bias may exist. However, the samples were generally representative of the Guangzhou population and we have weighted the results by population age and gender to reduce the influence of this possible bias. The comparison between the results of 2006 and 2013 surveys may partly reflect the different methods of data collection used in these two surveys, one using face-to-face interview (the 2006 survey) and one using telephone interview (the 2013 survey). However, in one local study similar methodological differences fail to produce different data [32], and so are an unlikely explanation for the large observed differences in behaviours and related perceptions in the two surveys. Furthermore, our study mainly focused on how the general public responded perceptually and behaviourally to the identification of H7N9 risk in Guangzhou. Future studies may investigate how change in human behaviours, such as poultry trading, will shape the ecology of H7N9 virus.

To conclude, live poultry markets and backyard poultry remain the two major sources of live bird exposures for the public in Guangzhou. Compared with their urban counterparts, rural residents were more likely to be exposed to live birds from sources other than LPMs, such as poultry farms, backyards and outdoors and conducted more risky practices such as touching when buying birds or slaughtering birds at home. The prevalence of buying live poultry from wet markets, touching when buying and slaughtering birds at home appears to have substantially declined among the Guangzhou population compared with the baseline measured in 2006. This may partly result from enhanced perceived risk of avian influenza being linked to live birds in LPMs. Therefore, although it is argued that the Chinese culture of buying live poultry from LPMs is difficult to change [33], we believe that promoting public awareness of the risk of avian influenza infection from exposure to live poultry in LPMs may gradually change people’s habits in the long term and subsequently lower the risk of avian influenza outbreaks. Geographic proximity to outbreaks could be an important factor associated with higher reduction in risky behaviours particularly reduction in buying live poultry from LPMs. Tracing the movement of poultry in the epidemic areas resulting from the control measures will also help to avoid spread of the virus. However, Zengcheng residents seemed to attribute the risk of H7N9 infection to live birds from wet markets only but not from other sources, such as backyard birds, suggesting limited awareness of, or perceptual bias regarding the role of live birds in disease transmission. The perceived likelihood of contracting H5N1 from buying live birds was lower than that of contracting H7N9 infection, possibly due to the greater number of reported H7N9 human cases in a short space of time in 2013. Risk communication should focus on reducing live bird exposure from two main sources (LPMs and backyards) and emphasize that being physically distant from the outbreak may not translate directly into reduced risk of H7N9 infection due to the interlinked poultry trading network. Specific risk communication strategies should also be implemented for rural residents who are more likely to resist change in habits of buying and handling live birds.

## Supporting Information

S1 Fig Monthly number of avian influenza A/H5N1 cases reported in China between August 2005 and July 2006. Source: World Health Organization (2015), Influenza at the human-animal interface: summary and assessment as of 1 May 2015. Available from http://www.who.int/influenza/human_animal_interface/Influenza_Summary_IRA_HA_interface_1_May_2015.pdf?ua=1.
(TIF)

S1 Table Questions and response scales for the study measures in the questionnaire. (DOCX)

S1 Dataset for behaviours related to live bird exposure and associated protective measures, Guangzhou, 2013. (CSV)

S2 Dataset for comparing risky behaviours and risk perception of avian influenza between the 2006 survey and the 2013 survey. (CSV)
