# Supplementary figures and images for: Live Bird Exposure among the General Public, Guangzhou, China, May 2013
Source: PLoS One. 2015 Dec 1;10(12):e0143582. doi: 10.1371/journal.pone.0143582 (PMC4666652; doi:10.1371/journal.pone.0143582)

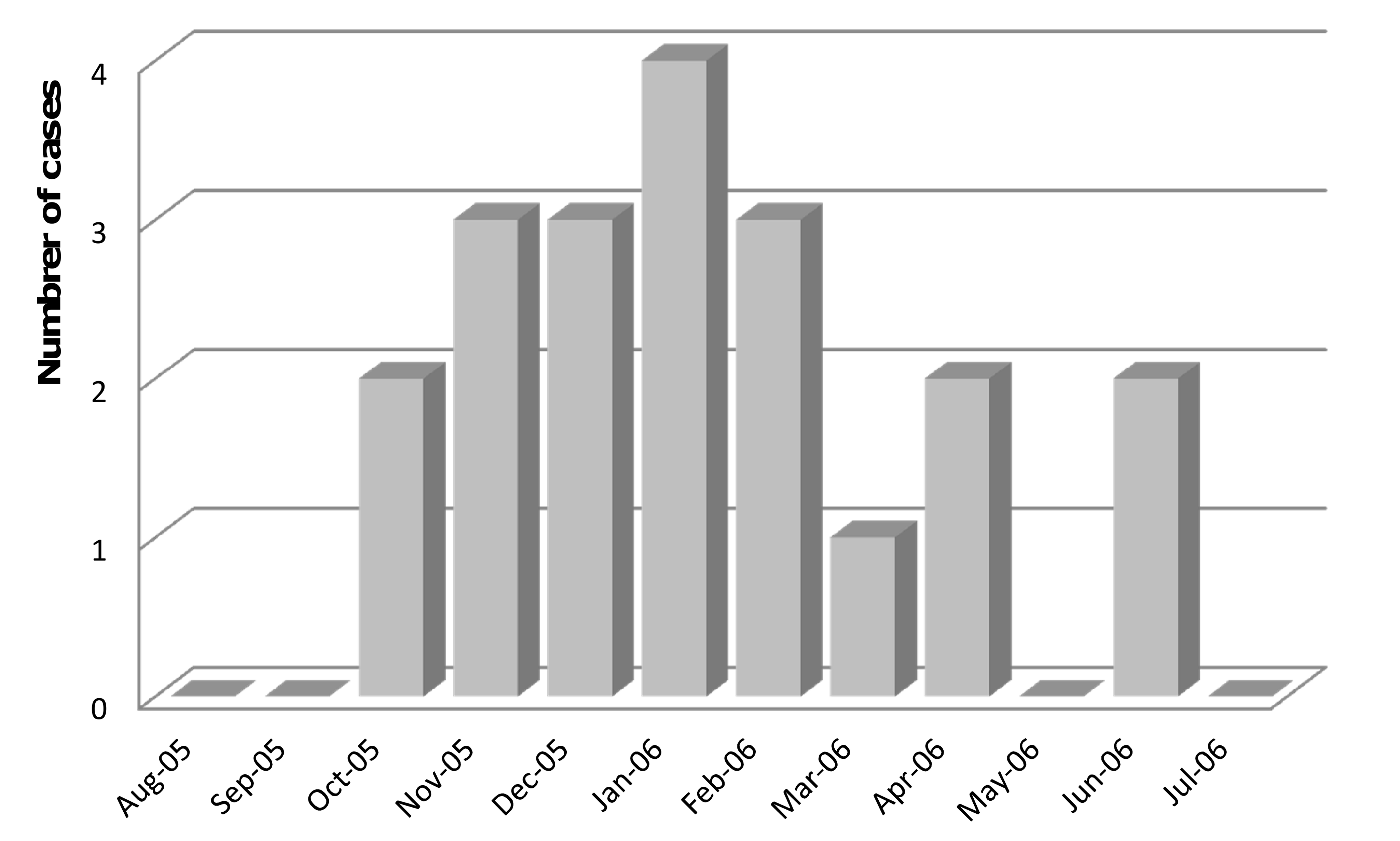

Supplement: S1 Fig — Source: World Health Organization (2015), Influenza at the human-animal interface: summary and assessment as of 1 May 2015. Available from http://www.who.int/influenza/human_animal_interface/Influenza_Summary_IRA_HA_interface_1_May_2015.pdf?ua=1. (TIF) [file pone.0143582.s001.tif]
