# Supplementary material for: Live Bird Exposure among the General Public, Guangzhou, China, May 2013
Source: PLoS One. 2015 Dec 1;10(12):e0143582. doi: 10.1371/journal.pone.0143582 (PMC4666652; doi:10.1371/journal.pone.0143582)
Supplement: S1 Table — (DOCX) [file pone.0143582.s002.docx]

**S1 Table.**  **Questions and response cales for the study measures in the questionnaire**

| Questions for the study measures | Response scale | Coded response |
| --- | --- | --- |
| How often did you visit wet markets to buy food over the past two months? | 1="never", 2="once", 3="around monthly", 4="2-3 times a month", 5="around weekly", 6="2-3 times a week", 7="4-6 times a week", 8="daily ", 9="more than once a day". | 1="never/once"  2="monthly or 2-3 times a month"  3="weekly or several times a week"  4="daily or more" |
| How often did you personally buy live chickens or other live birds from wet market over the past two month? | Same as above | Same as above |
| Over the past two months, did you buy live birds from other places other than wet markets? (if yes, where____) | 1="yes, once or more"  0="never" |  |
| Do you touch the live chicken or other poultry with your hand directly before you buy it? | 1="always", 2="usually", 3="sometimes", 4="never" | 1="always/usually/sometimes"  0="never" |
| When you buy live chickens or other poultry from a wet market do you kill and prepare them at home? | Same as above | Same as above |
| Over the past two months, did your family keep live chickens, duck, geese or other birds at home? | 1="yes"  0="no" |  |
| Did you have any contact with the birds you kept (including contact with birds, cages or feces of birds)? | Same as above |  |
| Over the past two months, did you contact with birds or their feces outdoors? | Same as above |  |
| Over the past two month, did you contact with sick or dead birds? | Same as above |  |
| (for those reported having some touching with birds when buying or touching backyard birds), Do you wash hands or use hand sanitisers/wiper as soon as possible after touching the birds | 1="always", 2="usually", 3="sometimes", 4="never" | 1="always/usually"  0="never/sometimes" |
| When killing birds at home, did you:  wear gloves  wear a face mask  wear aprons/outer garments/ coveralls  wear boots/boot covers | 1="yes"  0="no" |  |
| Have you changed your habit of buying live poultry after our government announced the H7N9 epidemic? | 1=yes, buy less  2=yes, never buy  3=yes, buy more (reasons:___)  4=no change |  |
